# Prevalence and Determinants of Postnatal Depression in Ernakulam District, India: A Cross-Sectional Study

**DOI:** 10.7759/cureus.74449

**Published:** 2024-11-25

**Authors:** Ani S Abraham, Sunu C Thomas, Jeby J Olickal, Kavumpurathu R Thankappan

**Affiliations:** 1 Public Health, Amrita Institute of Medical Sciences, Kochi, IND

**Keywords:** associated factors, india, kerala, loneliness, postnatal depression

## Abstract

Background

Most studies on postnatal depression (PND) in India are hospital-based, focusing on assessments within the first month of postpartum. Therefore, community-based studies are required to capture the full spectrum of PND. Factors associated with PND are required to be addressed to improve maternal and child health outcomes.

Objectives

We conducted this study to estimate the prevalence of PND and associated factors in one district of Kerala.

Methods

We conducted a community-based cross-sectional study among 330 postnatal women selected using multistage cluster sampling. A validated interview schedule was used to collect the data, which captured the socio-demographic details, obstetric factors, newborn characteristics, and individual and family-related factors. Information on PND was collected using the Edinburgh Postnatal Depression Scale. Log binomial regression analysis was done to find out the factors associated with PND.

Results

The mean (standard deviation) age of the mothers was 29.4 (4.89) years, and most were graduates (n=179, 54.2%). The prevalence of PND was 20% (95% confidence interval [CI] 16.04-24.65). Muslim mothers (adjusted prevalence ratio [APR] 2.07, CI: 1.18-3.41], scheduled caste/scheduled tribe (SC/ST) mothers (APR=2.49, CI: 1.22-5.09), those who had some stressful events during pregnancy (APR=2.05, CI: 1.11-3.77), and mothers experiencing loneliness (APR=5.83, CI: 3.84-8.87) were more likely to report PND than their counterparts.

Conclusion

About 20% of all the mothers reported PND. Prevalence was significantly higher among Muslim mothers, SC/ST mothers, and those experiencing prenatal stress or loneliness. Targeted community-based interventions for high-risk groups are the need of the time to reduce the prevalence of PND.

## Introduction

Postnatal depression (PND) is a treatable mental health condition with onset as soon as two weeks after childbirth and can remain indefinitely if left untreated [1,2]. It is classified in the Diagnostic and Statistical Manual of Mental Disorders, fifth edition (DSM-5), by the American Psychiatric Association as a significant depressive episode “with peripartum onset if mood symptoms emerge during pregnancy or within four weeks post-delivery [3].” Globally, maternal mental health issues are considered an important public health concern, with one in seven women being affected by it [4]. The prevalence of PND is reported to be 27.6% worldwide [5] and 20% in low and middle-income countries [6]. In India, the prevalence varied with the context, setting, and tool used, and a pooled prevalence of 20% was shown [7]. Untreated PND has negative effects on the mother’s quality of life and marital relationship, as well as it affects mother-child bonding. In spite of the public health importance of PND, up to 50% of PND remains unidentified [8]. Known risk factors for PND include psychosocial, obstetric, social, and interpersonal risk factors [9]. Additionally, mothers who experience loneliness may be more likely to develop PND than those who do not [10]. Persistence of PND has been documented as the cause of higher morbidity among women, particularly among those who remained unwell after six months [11].

Investigating PND within India's unique socio-cultural context is essential for identifying region-specific determinants of PND, thereby facilitating the development of evidence-based, culturally tailored interventions to enhance outcomes for both mothers and children. Several studies from India have identified various risk factors for PND. In the Indian context, factors such as gender preference of the newborn, poverty, intimate partner violence, and lack of social support are significant risk factors for the onset and persistence of PND [12]. Studies from South India have documented financial hardship, marital violence, lack of social support, delivery of a female child, and the effect of traditional rituals as risk factors for PND [12-14]. It was also found that family type, the occupations of women and their husbands, and poverty were independent indicators for PND, with rural mothers facing a particularly high risk after delivery [13]. In addition, factors such as caesarean sections, unexpected pregnancies, inadequate postpartum nutrition, and vitamin B12 deficiency were identified as contributing to PND. A study from Kolkata [15] showed that women living in nuclear families had a higher prevalence of PND. 

Most studies on PND in India are conducted in hospital settings, which limits their scope, as assessments typically occur within a month postpartum during follow-up visits, potentially overlooking cases that emerge later in the community. Community-based studies on PND are limited in the literature. Therefore, we conducted this study to determine the community-based prevalence of PND and its associated factors in a district of Kerala, India.

## Materials and methods

Study design and population 

A community-based cross-sectional study was conducted in the rural and urban areas of Ernakulam district, Kerala, among postnatal mothers who had delivered in the past 12 months before the study. Women who experienced stillbirth were excluded due to the sensitivity of the topic and the socio-cultural context of Kerala, where collecting such data might not be acceptable.

Sample size and sampling

**Figure 1:**
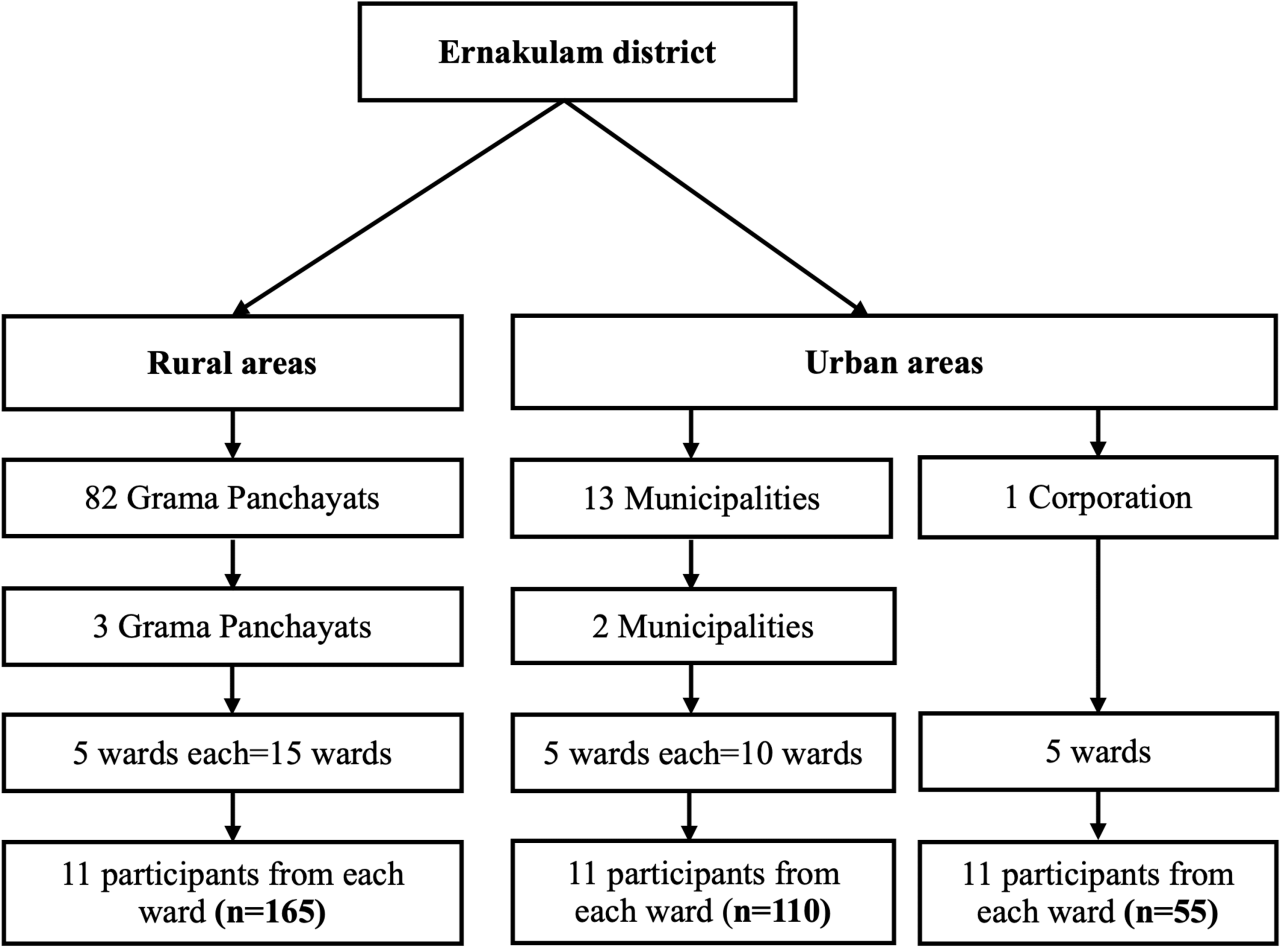
Flowchart illustrating the sample selection process Grama Panchayat: An institution of self-government for the rural areas.

Considering the prevalence of PND among mothers in the population as 31.4% [13], with a 95% confidence interval (CI), absolute precision of 6.28% (20% relative precision), and design effect of 1.5 to account for the cluster sample design, the sample size was estimated as 315. To recruit an equal number of participants across the 30 clusters (ward, the smallest administrative unit of the local government in Kerala), the sample size was rounded off to 330. A multistage cluster sampling technique was used to select the participants. A total of 30 clusters were selected, with 15 clusters in urban areas and 15 in rural areas. Every consecutive household with an eligible participant from the selected ward was recruited. From each cluster, 11 participants who met the eligibility criteria were recruited for the study. The sampling process is depicted in Figure 1.

Data collection and study tools

Data were collected from each participant at their homes. A validated interview schedule was used to collect the data, which captured the socio-demographic details, obstetric factors, newborn characteristics, and individual and family-related factors. PND was captured using the Edinburgh Postnatal Depression Scale (EPDS) [16], and loneliness was measured using the University of California Los Angeles (UCLA) loneliness scale version 3 [17]. The interview schedule was translated to Malayalam and back-translated to English till the meanings matched and was validated by experts in the field of Malayalam literature.

UCLA Loneliness Scale version 3 is a 20-item Likert scale with four responses to each item (often, sometimes, rarely, and never), which are scored from one to four, respectively. The total score ranges from 20 to 80. The severity of loneliness increases with the score. In this study, loneliness was categorized into four categories: no loneliness (20), mild (score 21-40), moderate (41-60), and severe (61-80) [18].

The EPDS was used for screening postnatal women for depression based on a one-week recall. It consists of 10 items; responses are scored as zero, one, two, or three according to the severity of symptoms. The total score is determined by adding the scores for each of the 10 items. The total score ranges from 0 to 30. The cut-off score is taken as 10, and those with scores more than 10 were considered to have symptoms of PND [19]. The cut-off scores for the EPDS and UCLA Loneliness Scale were chosen based on established guidelines and validation studies.

The questionnaire was administered face-to-face in a private setting to maintain confidentiality. Privacy was further ensured by conducting interviews in a space separate from family members or other individuals. 

Data analysis

The statistical analysis was conducted using Jamovi 2.3.28 and STATA version 14. Mean and standard deviation (SD) were computed for quantitative data. Categorical variables such as area of residence, education, religion, caste, occupation, socioeconomic status, type of family, total number of pregnancies, term of delivery, history of abortion, planned pregnancy, sex of the infant, intimate partner violence, stressful events during pregnancy such as the death or serious accidents of loved ones, and loneliness were summarized as frequencies and percentages. The prevalence of PND and loneliness was calculated with a 95% CI. A chi-square test was performed to find the factors associated with PND, and the unadjusted prevalence ratio (APR) with a 95% CI was calculated. A log-binomial regression was conducted for variables with a p-value <0.2 in the unadjusted analysis, and APRs with 95% CIs were calculated. A p-value <0.05 was considered statistically significant.

Ethical considerations

The study was reviewed and cleared by the Scientific Review Committee and Institutional Ethics Committee of Amrita Institute of Medical Sciences, Kochi (Approval number: ECASM-AIMS-2024-061) dated 30-01-2024. Written informed consent was obtained from all the participants before the interview.

## Results

Of the 337 participants approached for the study, seven declined to provide consent. All data were complete for the remaining participants, resulting in a total sample size of 330 used for the analysis. The sociodemographic characteristics of the study participants are summarised in Table 1. The mean (SD) age of the mothers was 29.4 (4.89) years. Most participants were graduates (n=179, 54.2%) and unemployed (n=233, 70.6%), with 29.7% (n=98) belonging to below the poverty line and 63.0% (n=208) in joint families. 

**Table 1:** Sociodemographic characteristics of mothers surveyed in Ernakulam district, Kerala (n=330) ^1^Others: students, government employee

Variables	Frequency (n)	Percentage (%)
Area of residence		
Rural	165	50.0
Urban	165	50.0
Education		
Postgraduation	67	20.3
Degree/diploma	179	54.2
Higher secondary	56	17.0
High school	28	8.5
Religion		
Hindu	126	38.2
Muslim	69	20.9
Christian	135	40.9
Caste		
General	139	42.1
Other backward caste	167	50.6
Scheduled tribe	4	1.2
Scheduled caste	20	6.1
Occupation		
Unemployed	233	70.6
Private job	76	23.0
Others^1^	21	6.4
Socioeconomic status		
Above poverty line	232	70.3
Below poverty line	98	29.7
Marital status		
Currently married	329	99.7
Widow	1	0.3
Type of family		
Nuclear	122	37.0
Joint	208	63.0

About 42% (n=138) had their first delivery, 20.9% (n=69) had a history of abortion, 51.5% (n=170) had caesarean sections, 34.8% (n=115) reported pregnancy-related comorbidities, and 8.2% (n=27) experienced delivery complications. Majority of the participants (n=207, 62.7%) reported mild loneliness postpartum. Nearly, one-third (32.7%, n=108) had newborns admitted to the NICUs (Table 2). 

**Table 2:** Maternal and child health characteristics of the study participants (n=330) *Five twin deliveries

Variables	Frequency (n)	Percentage (%)
Total number of deliveries		
One	138	41.8
Two	149	45.2
Three and above	43	13.0
Mode of delivery		
Vaginal	160	48.5
Caesarean	170	51.5
Term of current delivery		
Full term	318	96.4
Preterm	12	3.6
History of abortion	69	20.9
Complications during delivery	27	8.2
Planned pregnancy	284	86.1
Treatment for infertility	19	5.8
Intimate partner violence	3	0.9
History of mental illness	4	1.2
Stressful event during pregnancy	13	3.9
Infant birthweight in kg (n=335)*		
Normal weight	280	83.6
Low birth weight	55	16.4
Neonatal Intensive Care Unit admission		
Yes	108	32.7
No	222	67.3
Non-exclusive breastfeeding		
Yes	316	95.8
No	14	4.2
Difficulty during breastfeeding		
Yes	20	6.1
No	310	93.9
Loneliness		
Mild/no (scores 20-40)	276	83.6
Moderate/severe (scores >41)	54	16.4

The prevalence of PND was 20% (95% CI 16.04-24.65). The association of various factors with PND is depicted in Table 3. Muslim mothers were 2.07 times more likely to report PND than mothers who were Hindus (APR=2.07 95% CI: 1.18-3.41). Mothers who belonged to scheduled tribe/scheduled caste (ST/SC) were 2.49 times more likely to report PND than those in the general category (APR=2.49 95% CI: 1.22-5.09). Mothers with stressful events during pregnancy were 2.05 times more likely to have PND than those who didn’t have any stressful events during pregnancy (APR=2.05 95% CI: 1.11-3.77). Mothers who experienced loneliness were 5.83 times more likely to have PND than those who didn’t have loneliness (APR=5.83 95% CI: 3.84-8.87).

**Table 3:** Factors associated with postnatal depression: Results of bivariate and multivariable regression analysis (N=330) *Variables that had a p value <0.2 in the unadjusted analysis were used for the adjusted analysis. APL, above poverty line; APR, adjusted prevalence ratio; BPL, below poverty line; CI, confidence interval; OBC, other backward caste; PND, postnatal depression; SES, socioeconomic status; ST/SC, scheduled tribe/scheduled caste; UPR, unadjusted prevalence ratio

Variables	PND	No PND	UPR (95% CI)	APR (95% CI)	p-value
	n (%)	n (%)			
Area of residence					
Rural	30 (18.2)	135 (81.8)	1	-	-
Urban	36 (21.8)	129 (78.2)	1.2 (0.78-1.85)	-	-
Education					
Postgraduate	13 (19.4)	54 (80.6)	1.36 (0.48-3.81)	-	-
Degree/diploma	35 (19.6)	144 (80.4)	1.36 (0.53-3.56)	-	-
Higher secondary	14 (25)	42 (75)	1.75 (0.63-4.83)	-	-
High school	4 (14.3)	24 (85.7)	1	-	-
Religion					
Hindu	20 (15.9)	106 (84.1)	1	1	-
Muslim	22 (31.9)	47 (68.1)	2.01 (1.18-3.41)	2.07 (1.14-3.74)	0.015
Christian	24 (17.8)	111 (82.2)	1.12 (0.65-1.92)	1.14 (0.82-2.51)	0.194
Caste					
General	20 (14.4)	119 (85.6)	1	1	-
OBC	38 (22.8)	129 (77.2)	1.58 (0.96-2.58)	1.35 (0.80-2.29)	0.249
ST/SC	8 (33.3)	16 (66.7)	2.31 (1.15-4.64)	2.49 (1.22-5.09)	0.012
Occupation					
Unemployment	46 (19.7)	187 (80.3)	1.07 (0.62-1.84)	-	-
Private job	14 (18.4)	62 (81.6)	1	-	-
others	6 (28.6)	15 (71.4)	1.56 (0.68-3.54)	-	-
SES					
APL	50 (21.6)	182 (78.4)	1.32 (0.79-2.20)	-	-
BPL	16 (16.3)	82 (83.7)	1	-	-
Type of family					
Joint	44 (21.2)	164 (78.8)	1.17 (0.74-1.86)	-	-
Nuclear	22 (18.0)	100 (82.0)	1	-	-
Total number of pregnancies					
One	21 (19.1)	89 (80.9)	1.07 (0.58-1.92)	-	-
Two	30 (21.9)	107 (78.1)	1.21 (0.69-2.11)	-	-
Three and above	15 (18.1)	68 (81.9)	1	-	-
Term of delivery					
Full term	65 (20.4)	253 (79.6)	2.45 (0.37-16.22)	-	-
Preterm	1 (8.3)	11 (91.7)	1	-	-
History of abortion					
Yes	59 (85.5)	10 (14.5)	1.48 (0.78-2.74)	-	-
No	205 (78.5)	56 (21.5)	1	-	-
Planned pregnancy					
Yes	55 (19.4)	229 (80.6)	1	-	-
No	11 (23.9)	35 (76.1)	1.23 (0.70-2.18)	-	-
Sex of infant					
Male	124 (76.5)	38 (23.5)	1.41 (0.90-2.18)	1.35 (0.92-2.00)	0.122
Female	140 (83.3)	28 (16.7)	1	1	-
Intimate partner violence					
Yes	2 (66.7)	1 (33.3)	3.41 (1.48-7.81)	1.67 (0.70-4.00)	0.7
No	64 (19.6)	263 (80.4)	1	1	-
Stressful events during pregnancy					
Yes	7 (53.8)	6 (46.2)	2.89 (1.66-5.03)	2.05 (1.11-3.77)	0.021
No	59 (18.6)	258 (81.4)	1	1	-
Loneliness					
Mild/no	26 (9.7)	241 (90.3)	1	1	-
Moderate/severe	40 (63.5)	23 (36.5)	6.52 (4.33-9.82)	5.83 (3.84-8.87)	0.001

## Discussion

In our study, the prevalence of PND was comparable with findings from studies conducted in Ethiopia (23.7%), Tamil Nadu (19.8%) [20,21], and a systematic review conducted in India (22%) [7]. Sri Lanka, a country closer to Kerala with similar health indicators, reported a prevalence of 15% PND [22]. 

In this study, Muslim mothers were more likely to report PND than mothers of other religions. This finding is similar to a study conducted by Hanach et al. from the United Arab Emirates [23]. There is evidence for a relatively higher percentage of Muslim women who abstain from utilizing any contraceptive method due to religious convictions resulting in multiple pregnancies [24]. Furthermore, Muslim mothers experience elevated levels of maternal stress due to restrictions on their mobility outside the household during the postnatal period [25].

The caste of the mothers also emerged as a significant predictor for PND in this study. Mothers who were SCs/STs (the most backward caste in India) had a higher risk for PND than those in the general category. This is in contrast to other studies conducted in middle and northeast India and central India [26,27]. However, a study conducted in Rajasthan reported an association of PND with complications after delivery among the ST population [28]. Women from disadvantaged or marginalised communities face a compounded challenge where existing inequalities and social isolation exacerbate their experience of PND [29]. 

The study findings also showed that mothers who had stressful events during pregnancy had an increased risk of PND, which is similar to findings from other studies [7,30]. A dose-response relationship exists between stressful life events and the rate of PND. The accumulation of stressors might diminish maternal coping mechanisms and raise the probability of experiencing depression. Sufficient social support has the potential to mitigate the adverse impact of stressors, enabling improved emotional reactions and management of adverse situations. Consequently, this could help in preventing certain instances of PND and alleviating the intensity of symptoms in women who have already been diagnosed with PND [30].

In the current study, loneliness was significantly associated with PND, which is a new contribution to the current knowledge on PND [29]. Loneliness plays a role as an intermediate factor in the relationship between stigma associated with mental health and the presence of PND. Within the context of transitioning into motherhood, feelings of loneliness were perceived as a form of emotional detachment from both oneself and the infant, subsequently intensifying the levels of depression [29].

A single investigator collected the data, and there was consistency in data collection that avoided interobserver variability. However, the study had some limitations. The cross-sectional design of this study limits the ability to establish temporality, particularly for associations such as loneliness and PND. The interview was conducted at the residence of the participants, which might have resulted in their reluctance to respond to sensitive questions. There is a stigma surrounding mental health disorders, which might have resulted in under-reporting. This study did not capture the specific week or time period within the postnatal period when responses were collected. The reliance on a one-week recall period to identify PND may underestimate its prevalence and fail to capture the early or late onset of PND. Furthermore, the study's findings are specific to the population in Ernakulam district, limiting their generalizability to other regions.

## Conclusions

In our study, one in five mothers reported PND. Despite Kerala's high literacy rates and positive health indicators, there remains a critical need for early identification of risk factors and timely interventions to safeguard maternal mental health and the well-being of the mother-child dyad. PND screening should be integrated into regular postnatal follow-ups. Additionally, social interventions, such as peer support groups at the community level, can create a supportive environment where mothers can share their experiences and coping strategies. Incorporating Accredited Social Health Activist (ASHA) workers into interventions for postnatal mothers could be a promising approach, as their regular home visits provide an opportunity for early screening, mental health support, and linkage to healthcare services. Such community-based strategies could enhance the identification and management of PND effectively. Future research should focus on developing strategies to integrate screening for postnatal depression into routine healthcare practices. Furthermore, it is essential to address gaps in knowledge related to early and late-onset PND and concomitant medication for PND.
